# Supplementary material for: Blood meal analysis of tsetse flies ( Glossina pallidipes: Glossinidae) reveals higher host fidelity on wild compared with domestic hosts
Source: Wellcome Open Res. 2021 Aug 23;6:213. [Version 1] doi: 10.12688/wellcomeopenres.16978.1 (PMC8513123; doi:10.12688/wellcomeopenres.16978.1)
Supplement: Peer review history

# Peer review: Reviewer response for version 1

**DOI:** 10.21956/wellcomeopenres.18745.r46140

**Published:** 2021-10-12

**Authors:** Robert Opiro

**Affiliations:** 1 Department of Biology, Faculty of Science, Laroo Division, Gulu University, Gulu, Uganda

An interesting paper that tackles an important topic; identification of tsetse feeding preferences is important to the understanding of vector-host-pathogen interactions and their respective roles in the trypanosomiasis transmission cycle.  

 The authors used sequence analysis of the cytochrome b (cytb) gene to identify vertebrate host species in *Glossina pallidipes* tsetse blood meals to answer questions related to 1) the diversity of vertebrate hosts that flies fed on; 2) whether host feeding patterns varied in relation to type of hosts, tsetse feeding behaviour, site or tsetse age and sex; and 3) if there was a relationship between trypanosome detection and host feeding behaviours or host types.

 The study showed evidence of a wide range of hosts and multiple feeding episodes by individual flies but this was dependent on habitat and types of hosts there in. In wildlife-dominated areas, evidences of feeding on single hosts abound, while cases of mixed feed were more abundant in areas that had domestic hosts! The interpretation and conclusion by the authors in line with this observation is that perhaps domestic animals are not really preferred hosts by *Glossina pallidipes*, and this, the authors opine, could have important implications for understanding risk of transmission of trypanosomes between wildlife and livestock in interface areas.

 On the whole, this a well-written paper with technically and scientifically sound analyses techniques.

 My first argument with the authors would be why they used only cytb instead of COI, or both. I appreciate that they needed to compare their data with a previous study, and that cytb worked well in their preliminary analyses. But I would still have vouched for COI also because this would arguably have provided higher resolution of species identification compared to other gene targets like the cytochrome b used here. Moreover COI has the highest taxonomic coverage in reference databases. No wonder, a substantial number of flies (128) showed no evidence of a recent blood meal based on lack of amplification products following the screening with the Cb1 and Cb2 primers. Who knows, COI would have done better in this regard.

 Secondly, I would assume that the relative abundance of wild game, coupled with the areas with domestics animals, makes a wide choice of blood meals for tsetse but this would presumably vary with seasonal dynamics. Thus seasonal comparison of feeding preferences would be a nice direction for the authors to explore to answer some unresolved questions such as the absence of domestic cattle in the blood meals despite proximity to the same.

 There is also a statement that I disagree with in the introduction:  “Although there are multiple species of tsetse flies that can transmit multiple species of trypanosomes, ***Glossina pallidipes* is the most economically important species in East Africa**, because it is the main vector of Animal African Tryanosomiasis (AAT) and it is also a vector of Human African Trypanosomiasis (HAT)”. The authors cite a very old source (1972) for this assertion but I believe the dynamics have since changed making this statement highly debatable. For instance, in Uganda we currently know that *Glossina fuscipes fuscipes* is the most predominant and economically significant species as far as AAT and HAT transmission is concerned. So I find that statement a bit misleading; perhaps in Kenya it is but definitely not in the whole of East Africa.

Is the work clearly and accurately presented and does it cite the current literature?

Yes

If applicable, is the statistical analysis and its interpretation appropriate?

Yes

Are all the source data underlying the results available to ensure full reproducibility?

Yes

Is the study design appropriate and is the work technically sound?

Yes

Are the conclusions drawn adequately supported by the results?

Yes

Are sufficient details of methods and analysis provided to allow replication by others?

Yes

Reviewer Expertise:

My research interest is generally in the area of vectors and parasite biology including but not limited to population genetics and genomics, epidemiology and diagnostics of NTDs. The aim is to use these disciplines to improve our understanding of the distribution and movement of vectors and their parasites, with possible ramifications for the epidemiology of the diseases they are associated with.

I confirm that I have read this submission and believe that I have an appropriate level of expertise to confirm that it is of an acceptable scientific standard.

# Peer review: Reviewer response for version 1

**DOI:** 10.21956/wellcomeopenres.18745.r45586

**Published:** 2021-09-13

**Authors:** Boniface Namangala

**Affiliations:** 1 Department of Para-Clinical Studies, School of Veterinary Medicine, The University of Zambia, Lusaka, Zambia

The article Channumsin et al, titled "BLOOD MEAL ANALYSIS OF TSETSE FLIES (GLOSSINA PALLIDIPES: GLOSSINADAE) REVEALS HIGHER HOST FIDELITY ON WILD COMPARED WITH DOMESTIC HOSTS" is well thought, well written, with interesting data generated, that illustrate the significance of the tsetse fly as a vector that connects the sylvatic and domestic cycles of African trypanosomes which cause disease in livestock and humans.

 These authors, through sequencing of the mitochondrial cytochrome b gene and analyzed in relationship with previously determined trypanosome detection in the same flies, investigated (i) the diversity of vertebrate hosts that *Glossina pallidipes* flies from Kenya fed on; (ii) factors that determine host feeding patterns; and (iii) if there was a relationship between trypanosome detection and host feeding behaviours or host types. According to their data, in wildlife dominated areas, there was a much stronger tendency for flies to feed on single host species compared to sites where domestic hosts were more commonly fed on. In area including a mixture of resident domesticated animals, humans and wildlife, individual flies appeared to feed on multiple host species, suggesting that domestic animals are not the preferred hosts of the tsetse flies. These results suggest that individual flies in interface areas show higher feeding success on wild hosts when available but often feed on both wild and domesticated hosts.

 However, it is noteworthy that this study was only conducted during a specific season (July to August, 2012). It would be interesting to investigate how seasonality would influence the outcome of such a study.

Is the work clearly and accurately presented and does it cite the current literature?

Yes

If applicable, is the statistical analysis and its interpretation appropriate?

Yes

Are all the source data underlying the results available to ensure full reproducibility?

Yes

Is the study design appropriate and is the work technically sound?

Yes

Are the conclusions drawn adequately supported by the results?

Yes

Are sufficient details of methods and analysis provided to allow replication by others?

Yes

Reviewer Expertise:

Studies of neglected tropical diseases in Zambia, their epidemiology and control, with particular interest in human African trypanosomiasis. Surveillance and control of emerging and re-merging zoonoses. Surveillance and control of tick-borne diseases. Studies of immunological mechanisms governing resistance during the course of infection with protozoan parasites, with particular interest in human and animal trypanosome infections. Innate immune responses during protozoan infections, with particular emphasis on human and animal trypanosome infections.

I confirm that I have read this submission and believe that I have an appropriate level of expertise to confirm that it is of an acceptable scientific standard.
